# Modifiable Lifestyle Factors and Cognitive Function in Older People: A Cross-Sectional Observational Study

**DOI:** 10.3389/fneur.2019.00401

**Published:** 2019-04-24

**Authors:** Noriyuki Kimura, Yasuhiro Aso, Kenichi Yabuuchi, Masato Ishibashi, Daiji Hori, Yuuki Sasaki, Atsuhito Nakamichi, Souhei Uesugi, Hideyasu Fujioka, Shintaro Iwao, Mika Jikumaru, Tetsuji Katayama, Kaori Sumi, Atsuko Eguchi, Satoshi Nonaka, Masakazu Kakumu, Etsuro Matsubara

**Affiliations:** ^1^Department of Neurology, Faculty of Medicine, Oita University, Oita, Japan; ^2^TDK Corporation, Tokyo, Japan

**Keywords:** cross-sectional study, lifestyle factors, cognitive function, wearable sensor, mini-mental state examination, random forest regression analysis

## Abstract

**Background:** The development of evidence-based interventions for delaying or preventing cognitive impairment is an important challenge. Most previous studies using self-report questionnaires face problems with reliability and consistency due to recall bias or misclassification among older people. Therefore, objective measurement of lifestyle components is needed to confirm the relationships between lifestyle factors and cognitive function.

**Aims:** The current study examined the relationship between lifestyle factors collected with wearable sensors and cognitive function among community-dwelling older people using machine learning.

**Methods:** In total, 855 participants (mean age: 73.8 years) wore a wristband sensor for 7.8 days on average every 3 months. Various lifestyle parameters were measured, including walking steps, conversation time, total sleep time (TST), sleep efficiency, time awake after sleep onset, awakening count, napping time, and heart rate. Random forest (RF) regression analysis was used to examine the relationships between total daily sensing data and Mini-Mental State Examination (MMSE) scores. Confounding factor analysis was conducted with models that were adjusted and unadjusted for demographic and vascular risk factors, and selected variables were assessed as risk and protective factors using partial dependence plots (PDPs).

**Results:** Lifestyle data were collected for 31.3 ± 7.1 days per year using wristband sensors. RF regression analysis adjusted for age, gender, and education levels selected four variables, including number of walking steps, conversation time, TST, and heart rate. Moreover, walking steps, conversation time, and heart rate remained after RF regression analysis adjusted for demographic and vascular risk factors. Number of walking steps, conversation time, and heart rate were categorized as protective factors, whereas TST was categorized as a risk factor for cognitive function. Although PDPs of number of walking steps and heart rate revealed continuously increased MMSE scores, those of conversation time and TST and revealed that the tendency in the graph was reversed at the boundary of a particular threshold (321.1 min for conversation time, 434.1 min for TST).

**Conclusions:** Lifestyle factors, such as physical activity, sleep, and social activity appear to be associated with cognitive function among older people. Physical activity and appropriate durations of sleep and conversation are important for cognitive function.

## Introduction

Dementia is a major public health issue worldwide, with a serious burden for patients, caregivers, and society, as well as substantial economic impacts (1). Although the prevalence of late-life cognitive impairment and dementia are expected to increase in future, effective disease-modifying treatments are currently unavailable. Therefore, understanding the modifiable risk factors and developing evidence-based interventions for delaying or preventing cognitive impairment is an important challenge. Numerous observational studies have reported a range of potentially modifiable risk factors for dementia, including lower levels of education, midlife hypertension, midlife obesity, diabetes mellitus, smoking, and late-life depression, as well as social isolation, physical inactivity, and hearing loss (2–6). Depression, physical inactivity, and social isolation are particularly important predictors of late-life cognitive impairment (4, 7). Sleep disturbance is also prevalent among older people, representing a risk factor for cognitive impairment (8–11). However, most previous studies have used self-report questionnaires, which can have problems with reliability and consistency due to recall bias or misclassification, particularly among older people, or those with mild cognitive impairment (12–15). Moreover, physical activity questionnaires are not able to capture non-exercise physical activity, which accounts for most total activity energy expenditure among older people and social relationship questionnaires regarding social network size or social engagement cannot accurately measure the duration of contact with family members or friends (16). Therefore, objective measurement of lifestyle components is needed to confirm the relationships between lifestyle factors and cognitive function. Recently, wearable sensors have been used to evaluate lifestyle factors such as physical activity and the sleep-wake cycle in large epidemiological studies (12–15, 17–21). Wearable sensors are non-invasive and cost-effective, and can record total daily movement and the sleep-wake cycle continuously and objectively 24 hours/day without recall bias. In the present study, we developed a wristband sensor enabling quantification of the conversation time for assessing social contact in addition to physical activity and the sleep-wake cycle. Moreover, random forest (RF) regression analysis was conducted to identify risk and protective factors of the lifestyle components associated with Mini-Mental State Examination (MMSE) scores. RF is an ensemble learning method for classification, regression and other functions, which operates by constructing a multitude of decision trees at training time, and outputs the class that is the mode of the classes or mean prediction of the individual trees (22). Machine learning techniques can shorten the time required for big data analysis, and can identify patterns in complex scenarios that are impossible for humans to identify (23). Therefore, machine learning has been applied in disease diagnosis, development of prediction models and identification of risk factors (23–26). The current study aimed to examine the relationship between lifestyle factors collected by wearable sensors and MMSE scores in community-dwelling older people using machine learning.

## Methods

### Participants

**Figure 1:**
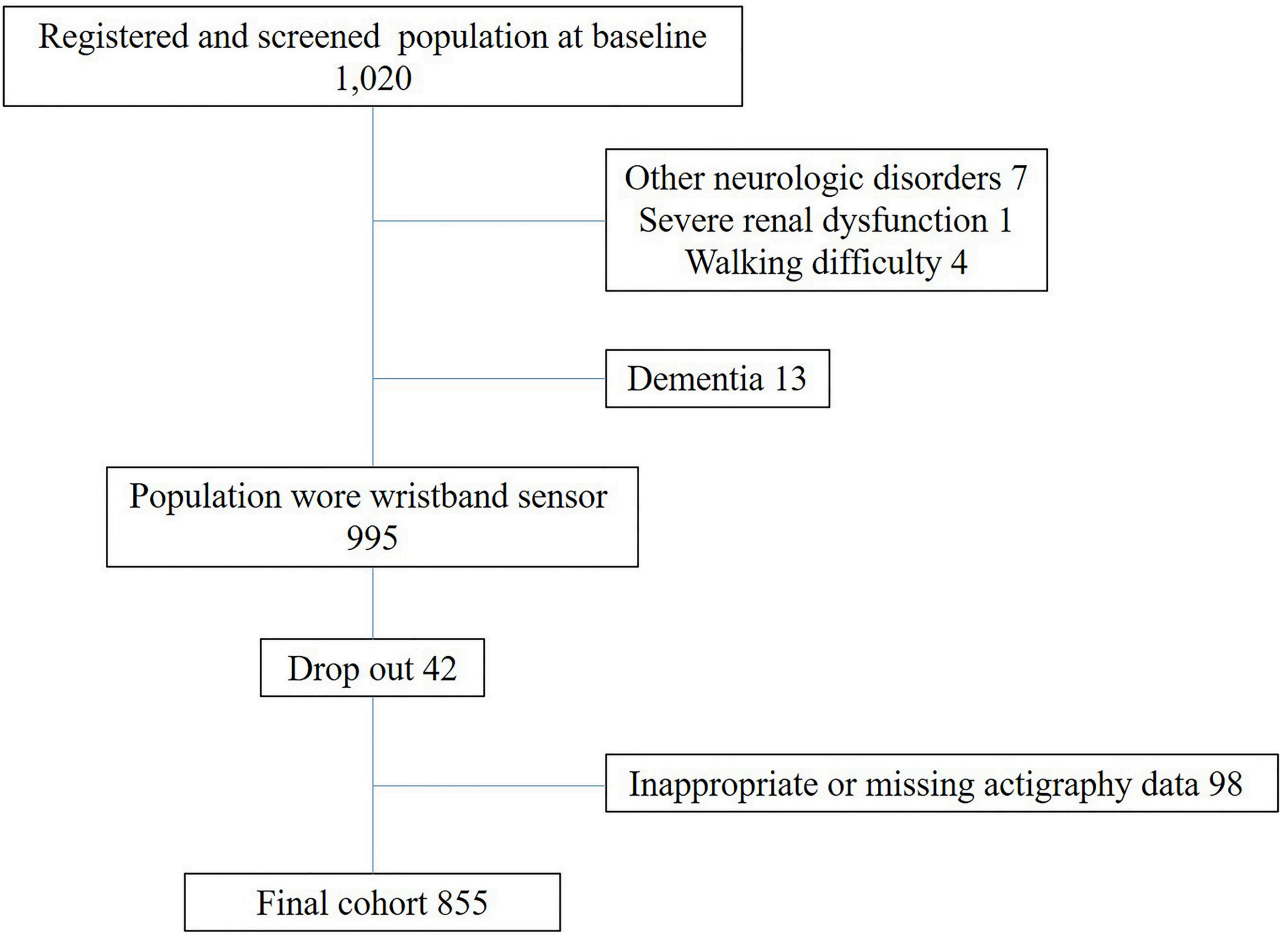
Flow of participant recruitment.

We have been conducting a community-based observation study focusing on lifestyle risk and preventive factors related to dementia in Usuki city, in southern Japan, since 2015. The proportion of the population over 65 years old in Usuki city has reached 38%, compared with 27.3% of the nationwide population in Japan. In the present study, public servants carried out public relations initiatives to recruit participants aged 65 or older without dementia from the entire city using electronic and paper-based media because the lifestyle factors such as physical activity and social isolation are closely related to late-life cognitive impairment (1, 7). From August 2015 to March 2016, a total of 1,020 community-dwelling people agreed to participate in our prospective cohort study examining risk and protective lifestyle factors for dementia among older people. For inclusion, participants met the following criteria: (1) 65 years and older; (2) living in Usuki city; (3) healthy physical and psychological condition; (4) MMSE score 20 points or more and absence of dementia diagnosis or administration of dementia medication; (5) independent function in activities of daily living. The exclusion criteria included a history of other neurological and psychiatric disorders including Parkinson's disease or epilepsy, severe head trauma, alcoholism, severe cardiac failure, and severe hepatic or renal dysfunction, undergoing treatment for cancer, and walking difficulty due to stroke sequelae. All participants underwent a physical examination, evaluation of cognitive function and medical interview at baseline. Height and weight were measured and body mass index (BMI) was calculated as weight in kilograms divided by height in m^2^. We collected information about demographic characteristics, including age, gender, education level, and smoking status as well as alcohol consumption and medication history via interviews conducted by trained medical staff at baseline. Moreover, history of chronic disease was defined as a prior diagnosis of stroke, cardiac disease, and hepatic or renal disease as well as cancer. Assessment of vascular risk factors, such as hypertension, diabetes mellitus, and hypercholesterolemia were based on a detailed clinical history and information of medicine (antihypertensive, antidiabetic, or anticholesterol medication). Because the MMSE is widely used for dementia screening tool, cognitive function was evaluated using the MMSE. The results of MMSE were reviewed by neurologist and clinical psychologist for the primary screening for dementia. Participants were considered to have possible dementia was when they scored <20 points on the MMSE (27). Moreover, we collected the further information regarding dementia diagnosis or administration of dementia medication in the local hospital, and daily living decline due to cognitive impairment from participants and their closest relatives in the face-to-face clinical interview. Diagnosis of dementia was made according to the criteria of the Diagnostic and Statistical Manual of Mental Disorders-5 (28) by a neurologist who used all cognitive and clinical data. Of 1,020 participants, seven participants had other neurological disorders, and one participant had severe renal dysfunction. Four participant had difficulty walking without assistance. Although we recruited participants without dementia via electronic and paper-based media, 13 participants with dementia were identified based on interviews at the first examination. A total of 25 participants with other neurological disorders, severe renal dysfunction, difficulty walking, or dementia were excluded from the present study. The remaining 995 participants were asked to wear a wristband sensor on the wrist for 7–14 days on average every 3 months (total study period 56 days). To eliminate measurement error due to seasonal differences in lifestyle because previous studies using actigraphy measured physical activity and sleep data for a maximum of 3 days (14, 20). Therefore, average annual data were used to examine the relationships between lifestyle factors and cognitive function. A total of 42 participants refused to wear the wristband sensor during the first cycle, and 98 participants had inadequate sensing data for analysis. Thus, the final sample consisted of 855 participants (317 men and 538 women, mean age 73.8 ± 5.8 years, education years 11.8 ± 2.1) with cognitive assessment and valid sensing data (Figure 1). The mean age of our participants was rather high, which might reflect the increasing population aging rate in Usuki city. Similarly, previous studies investigated the relationship between sleep or physical activity and cognition in the very elderly people (13, 21). The excluded participants had 1.7 year older (75.5 ± 6.9 years, *p* = 0.0097), slightly lower education years (11.3 ± 1.7, *p* = 0.0076), and lower MMSE scores (median 28, *p* = < 0.0001) than 855 participants who were included in our analysis. However, two groups did not differ in the gender distribution (42 men and 98 women, *p* = 0.1061), smoking states (ever smoker 7.4%, *p* = 0.1338), alcohol consumption (ever drinker 38.9%, *p* = 0.6168), and history of chronic diseases (hypertension 55.8%, *p* = 0.2651, diabetes mellitus 13.4%, *p* = 0.9861, hypercholesterolemia 27.3%, *p* = 0.2374, respectively). This prospective study was conducted in accordance with the Declaration of Helsinki, and was approved by the local ethics committee at Oita University Hospital (UMIN000017442). Written informed consent to participate in the study was obtained from all participants.

### Wearable Sensor Data

All participants were asked to wear a wristband sensor (Silmee™ W20, TDK Corporation Tokyo, Japan) on the wrist, except while bathing. We excluded data if the heart rate count indicated that the wristband sensor had been removed. Sixteen of 98 participants who had inadequate sensing data (16.3%) did not wear wristband sensor during sufficient period for analysis. Our wearable sensor measured various lifestyle parameters, including heart rate, walking steps, conversation time, total sleep time (TST), sleep efficiency, time awake after sleep onset (WASO), awakening count, and napping time. These parameters were calculated by summing sensing data each day and averaging this over the whole measurement period.

#### Physical Activity

Physical activity data were detected by a 3-axis accelerometer, which enabled measurement of acceleration in three perpendicular axes. The evaluation circuitry converted the output of a micromechanical acceleration-sensing structure, according to the differential capacitance principle. The accelerometer generated physical activity data regarding walking steps with composite acceleration of 3-axis measurement every time the wearable sensor was moved, and data were captured continuously and summarized in 1 min intervals. Walking steps were identified by capturing frequency bands ranging from 2 to 3 Hz, which synthesized acceleration by the accelerometer. The number of walking steps was calculated by summing the number of steps for each day and dividing this by the number of days of lifestyle data measurement. Therefore, the number of walking steps was represented as the average number of steps per day.

#### Sleep

**Figure 2:**
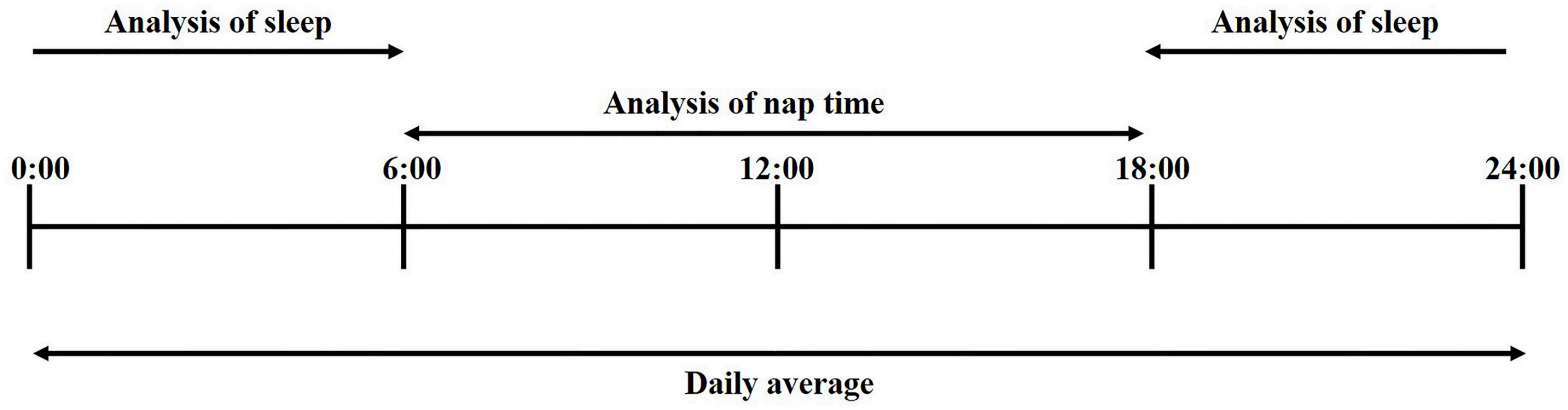
Indices of wearable sensor.

Sleep-wake parameters were assessed by the magnitude of synthesized acceleration of the 3-axis accelerometer and cumulative energy. The data were confirmed and adjusted by qualified technicians using visual inspection. Time in bed between bedtime and waking time was determined by the activity count recorded by the wristband sensor. Sleep parameters such as TST, WASO, and sleep efficiency, as well as the awakening count were measured from 18:00 in the evening to 5:59 the following morning (Figure 2). Sleep Start was defined as the clock time associated with the beginning of the first 20-min block of sleep without movement (20, 21). TST was defined as the average total number of minutes slept per day. Sleep fragmentation was evaluated by WASO, sleep efficiency, and awakening count. Nocturnal awakening was defined as 20 min of continuous movement from sleep onset to the end of sleep (20, 21). Therefore, WASO and awakening count were calculated by averaging the total number of minutes awake and the number of minutes of sleep per day. Sleep efficiency was calculated as the percentage of TST over the time spent in bed. Although a sleep dairy was not used in this study, the total time in bed between bedtime and getting up was determined by TST and WASO. Nap time was defined as resting without movement on the wearable sensor from 6:00 in the morning to 17:59 in the evening (Figure 2).

#### Heart Rate

Heart rate was detected by photoplethysmography. Pulse photoplethysmography is a simple and useful method for monitoring heart rate. Using this method, pulse measurement is based on the irradiation of 573 nm wavelength light, and the conversion of the intensity of reflected light to an electrical signal. The heart rate was calculated by summing pulses per min for each day and dividing this by the number of days in the lifestyle data measurement period. Therefore, heart rate was represented as the average number of pulses per day.

#### Conversation Time

Our wearable sensor could not detect the content of conversation, but could detect utterances of the participant wearing the wearable sensor, and utterances of nearby people. Sound data were captured continuously during the presence or absence of a conversation every minute. Although the utterances of other individuals were included in the sound data, participating in the conversation was considered to be important for social activity in this study.

##### Principle of detection

Sound data were collected by a microphone on the wearable sensor, and analyzed to evaluate the conversation time. Our wearable sensor detected sound pressure, which was produced by utterance within a 2-meter radius from the device. The sound pressure range was from 55 to 75 dBA at this distance. The conversation time was defined as the frequency components included in conversation data extracted by signal processing. In detail, the wearable sensor extracted a frequency band corresponding to a human voice from the sound data within the sound pressure range as a sound frame. Conversation was defined as more than four sound frames per minute, during a 1-min period. It is possible that the sound of television viewing or radio listening was detected as conversation due to the detection method based on the sound pressure and frequency. Therefore, we also quantified the detection rate of television viewing.

#### Verification of Detection Accuracy

##### Physical activity

The accuracy of walking step detection was verified by comparing the sensing data and video observation data. Walking steps were simultaneously collected for 9 min by wristband sensor and continuous video monitoring in twenty healthy participants aged 60–80 years (11 men and 9 women). Significant correlation was found between walking steps measured by wristband sensor and those from video observation (*r* = 0.9869, *p* < 0.0001, Pearson correlation, Supplemental Figure 1).

##### Sleep detection

The accuracy of sleep duration detection was verified by comparing the sensing data and video observation data. Sleep duration was simultaneously collected during night time by wristband sensor and continuous video monitoring in five healthy participants aged 20–60 years (5 men). Significant correlation was found between sleep duration from wristband sensor and that from video observation (*r* = 0.9995, *p* < 0.0001, Pearson correlation, Supplemental Figure 2).

##### Conversation time

The accuracy of conversation time detection was verified by comparing the sensing data and self-report data regarding conversation time. Sound data were captured for 50 h in healthy participants aged 30–40 years and analyzed in terms of precision, recall, and F-Measure (Supplemental Table 1). The results revealed values of 0.698 for precision, 0.774 for recall, and 0.734 for F-Measure. The false detection rate was calculated to evaluate the false detection of sounds other than conversation, such as television, noise during commuting, or noise during office work. The results of the false detection rate analysis are shown in Supplemental Table 2. Furthermore, we verified the false detection rate of sounds, including clothing noise, wind, breath, train, motor vehicles, guitar, piano, violin, cat, dog, bird, vacuum cleaner, tooth brushing, washing machine, dishwasher, and dish-washing, which were likely to be erroneously detected as a conversation. We adjusted each sound to a 55–75 dBA sound pressure range in front of the microphone on the wearable sensor, and input each sound for 100 min continuously to verify the false detection rate. The total false detection rate in the same sound pressure environment was 4.5% (sum of each time)/(number of items ^*^100 min). These results indicate that the conversation time detected by the wearable sensor could be used as a reliable indicator of human conversation time. Moreover, the accuracy of conversation time detection in twenty healthy participants aged 60–80 years was verified by comparing the sensing data and video observation data. Conversation time was simultaneously collected for 9 minutes by wristband sensor and continuous video monitoring in twenty healthy participants aged 60–80 years (11 men and 9 women). Significant correlation was found between conversation time from wristband sensor and that from video observation (*r* = 0.8512, *p* < 0.0001, Pearson correlation, Supplemental Figure 3).

### Statistical Analysis

RF regression analysis was used to examine the relationships between total daily sensing data and MMSE score. RF is an ensemble learning method that operates by constructing decision trees using bootstrap aggregation, and computes node impurity for every variable. This analysis can be used to rank the variables based on their predictive importance, such as %IncMSE and IncNodePurity. Moreover, confounding factor analysis was performed to build an unadjusted model (model 0), a model adjusted for demographic factors (model 1), and a model adjusted for demographic and vascular risk factors (model 2). The selected variables were used to assess the risk and protective factors for cognitive function using partial dependence plots (PDPs).

#### RF Regression Analysis

**Figure 3:**
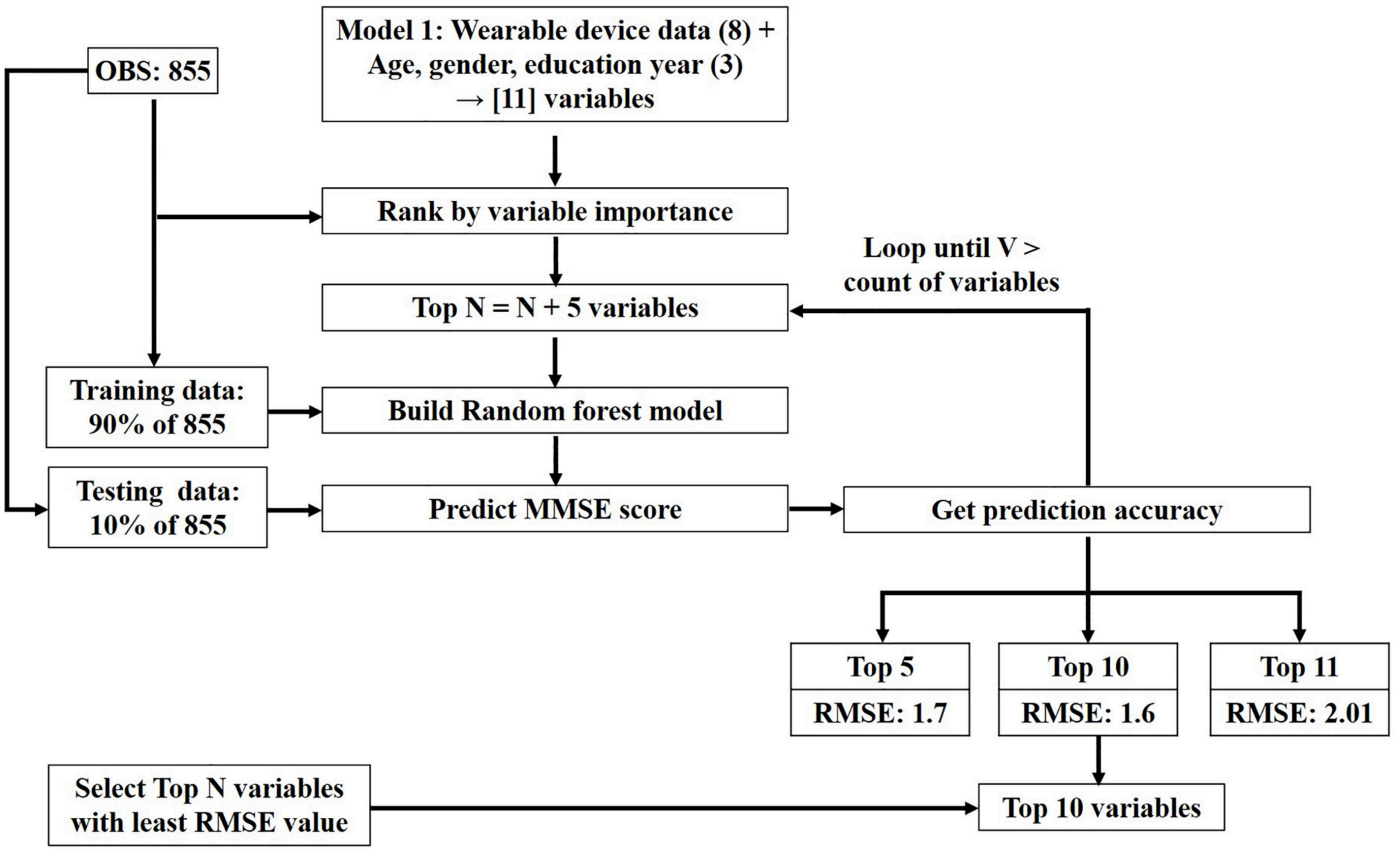
Top N variables selection process.

RF was conducted using R (version 3.4.1) and an RF package for Windows 10 (6, 22, 29, 30). The inbuilt bootstrap aggregation procedure of the RF algorithm enables the learning algorithm to be limited to a random sample of features to search. This drastically reduces the variance and avoids the problem of overfitting. The tree was grown on a bootstrap sample (“the bag”) by placing two-thirds of the cases in the bag and the remaining one-third “out-of-bag” (OOB) (28). Default values of 500 for the ntree (a hyper parameter to define the number of trees to grow in the model) and p/3 (p indicates the number of predictors) for mtry (a hyper parameter used by the algorithm to determine the count of variables to be randomly sampled for search during each split point) were set. The p/3 value is the default mtry value recommended by the algorithm inventors. The hyper parameters ntree and mtry were not tuned for the variable selection process. Assessment of variable importance was performed using IncNodePurity (a variable importance measure). High IncNodePurity values indicated high importance. A total of 855 samples were used to rank the variables by importance. The selection of top N variables was determined by prediction performance of a model built using 90% of the total 855 samples, assessed by the root mean square error (RMSE) using 10% of the total 855 samples. By applying this rule, the top N variables were selected for which the RMSE value was lowest (Figure 3). Table 1 shows all 17 variables. The adjusted model 1 used 11 variables, including age, gender, education year, and eight wearable sensor variables, whereas the adjusted model 2 used 17 variables for the variable selection process in the RF regression analysis using daily sensing data.

**Table 1:** All variables for RF analysis.

**Variables**
Age (years)
Gender (0; Male, 1; Female)
Education (years)
BMI (kg/m^2^)
Smoking status (0; Every day, 1; None, 2; Sometimes)
Alcohol consumption (0; Every day, 1; None, 2; Sometimes)
Hypertension (0; No, 1; Yes)
Diabetes mellitus (0; No, 1; Yes)
Hypercholesterolemia (0; No, 1; Yes)
Walking steps (steps/day)
Conversation time (mins/day)
Heart rate (counts/mins/day)
TST (mins/day)
WASO (mins/day)
Sleep efficiency (%/day)
Awakening time count (counts/day)
Nap time (mins/day)

TST, Total sleep time; WASO, time awake after sleep onset, mins; minutes

#### Confounding Factor Analysis

Potential confounding factors included age, gender, education levels, and BMI, alcohol consumption, smoking status, and vascular risk factors, which may affect both lifestyle factors and cognitive function (6). Therefore, the present study used RF regression analysis unadjusted (model 0), adjusted for age, gender, and education year (model 1), and adjusted for all confounding factors (model 2). Multiple linear regression analysis (R version 3.4.1 for Windows 10) was used to identify the effects of confounders and adjust for potentially confounding variables in the model (31). The confounding analysis procedure was conducted in R (30). Multiple linear regression models were built for models 0, 1, and 2 to identify the variables influenced by the confounding factors. The regression coefficients of independent variables were calculated in models 0, 1, and 2 (Table 2). The confounding effects on the independent variables were measured by the percentage changes in the estimated regression coefficients as follows: 100 × (adjusted coefficient—unadjusted coefficient)/(unadjusted coefficient). The influenced independent variables were defined as the conditions in which regression coefficient values in model 1 and 2 were increased by more than 200%, decreased by more than 200%, or cases in which the independent variable sign was reversed or independent variable was newly added compared with the model 0. Finally, the independent variables influenced by confounding factors were excluded from the adjusted model 1 and 2.

**Table 2:** Confounding factors in model 0, 1, and 2.

**Model 0**	**Model 1**	**Model 2**
Unadjusted	Adjusted for age, gender, education year	Adjusted for age, gender, education year, BMI, hypertension, diabetes mellitus, hypercholesterolemia, alcohol consumption, smoking status

*BMI, Body mass index*.

#### Risk and Protective Factor Analysis

The variables in model 1 identified by RF and confounding factor analysis were assessed for risk and protective factor analysis. In black box methods like RF analysis, functional relationships between each independent variable and the response variable are assessed using PDP (32). PDP is a simple technique for visualizing partial relationships between the outcome and the predictors. PDP enables visualization of relationships between y and one or more predictors, xj, as detected by RF analysis. In this method, xj is the predictor of interest, X–j represents the other predictors, y is the outcome, and ^∧^f(X) is the fitted forest. The partial dependence algorithm functions as follows:

1. For xj, sort the unique values V = {xj}iϵ{1,…,n} resulting in V*, where |V*| = K. Create K new matrices X(k) = (xj = V*k, X–j), ∀k = (1,…,K).
2. Drop each of the K new datasets, X(k) down the fitted forest resulting in a predicted value for each observation in all K datasets: ^∧^y(k) = ^∧^f(X(k)), ∀k = (1,…,K).
3. Average the predictions in each of the K datasets, ^∧^y* k = 1 nPN I = 1 ^∧^y (k) i, ∀k = (1,…,K).
4. Visualize the relationship by plotting V* against ^∧^y*.

PDPs are generated from the RF model and used to define the variables as risk and protective factors for MMSE scores. The marginal prediction data were extracted from the PDP for all the variables in the RF model. The correlation value was calculated between each independent variable and MMSE score, using the marginal prediction data. Variables with positive correlation values were defined as protective factors, whereas those with negative correlation values were defined as risk factors. Moreover, PDPs for each selected variable were used to determine risk and protective factors.

#### Contribution and Significance of Risk and Protective Variables

The variables in model 1 identified by RF and confounding factor analysis were applied in multiple linear regression for quantifying the contributions of individual risk and protective variables. ^*^P < 0.05 were considered statistically significant.

#### Model Prediction Accuracy

Moreover, the model prediction accuracy was verified by linear regression and RF analyses. The training models, including linear regression and RF algorithms, were built using the data set with the variables walking steps, conversation time, TST, heart rate, age, gender, and years of education, and were evaluated with the test data. The performance of the model was evaluated by the prediction RMSE with the lowest value. We used 90% of the 855 samples for training, and 10% of the 855 samples for testing.

## Results

### Clinical and Demographic Characteristics of Participants and Wristband Sensor Data

Table 3 summarizes the sociodemographic factors, cognitive function, and lifestyle factors of all participants. Participants' mean age was 73.8 years, and 62.9% of participants were female. Lifestyle data were collected from participants for 31.3 ± 7.1 days per year (7.8 days on average every 3 months) using the wristband sensor.

**Table 3:** Summary of demographic characteristics and wearable sensor data of participants.

**CHARACTERISTICS**	
Age (years)	73.8 ± 5.8
Gender (M:F)	317:538
Education (years)	11.8 ± 2.1
BMI (kg/m^2^)	23.2 ± 3.1
Median MMSE scores	29 (20, 30)
Ever smoker	36 (4.2%)
Ever drinker	354 (41.4%)
**PAST HISTORY**	
Hypertension	429 (50.2%)
Diabetes mellitus	114 (13.3%)
Hypercholesterolemia	281 (32.9%)
**WEARABLE SENSOR DATA**	
Walking steps (steps/day)	5452.9 ± 2778.0
Conversation time (mins/day)	219.7 ± 86.3
Heart rate (counts/mins/day)	64.7 ± 6.3
TST (mins/day)	408.4 ± 69.1
WASO (mins/day)	22.1 ± 14.1
Sleep efficiency (%/day)	1.0 ± 0.0
Awakening time count (counts/day)	0.5 ± 0.3
Nap time (mins/day)	48.7 ± 39.3

*M, Male; F, Female; BMI, Body mass index; MMSE, Mini-Mental State Examination; TST, Total sleep time; WASO, time awake after sleep onset; (20, 30), the range of MMSE value is from 20 to 30; mins, minutes*.

### RF Regression Analysis Using Daily Sensing Data

#### Selecting Important Variables

RF regression analysis using sensing data revealed that the five variables (walking steps, conversation time, TST, and WASO as well as heart rate) in model 0 were selected. Moreover, the top 10 variables (walking steps, conversation time, TST, and WASO, sleep efficiency, awakening time count, naptime, heart rate as well as age and education years) in model 1 were selected based on the lowest RMSE value (1.6).

#### Confounding Factor Analysis

**Figure 4:**
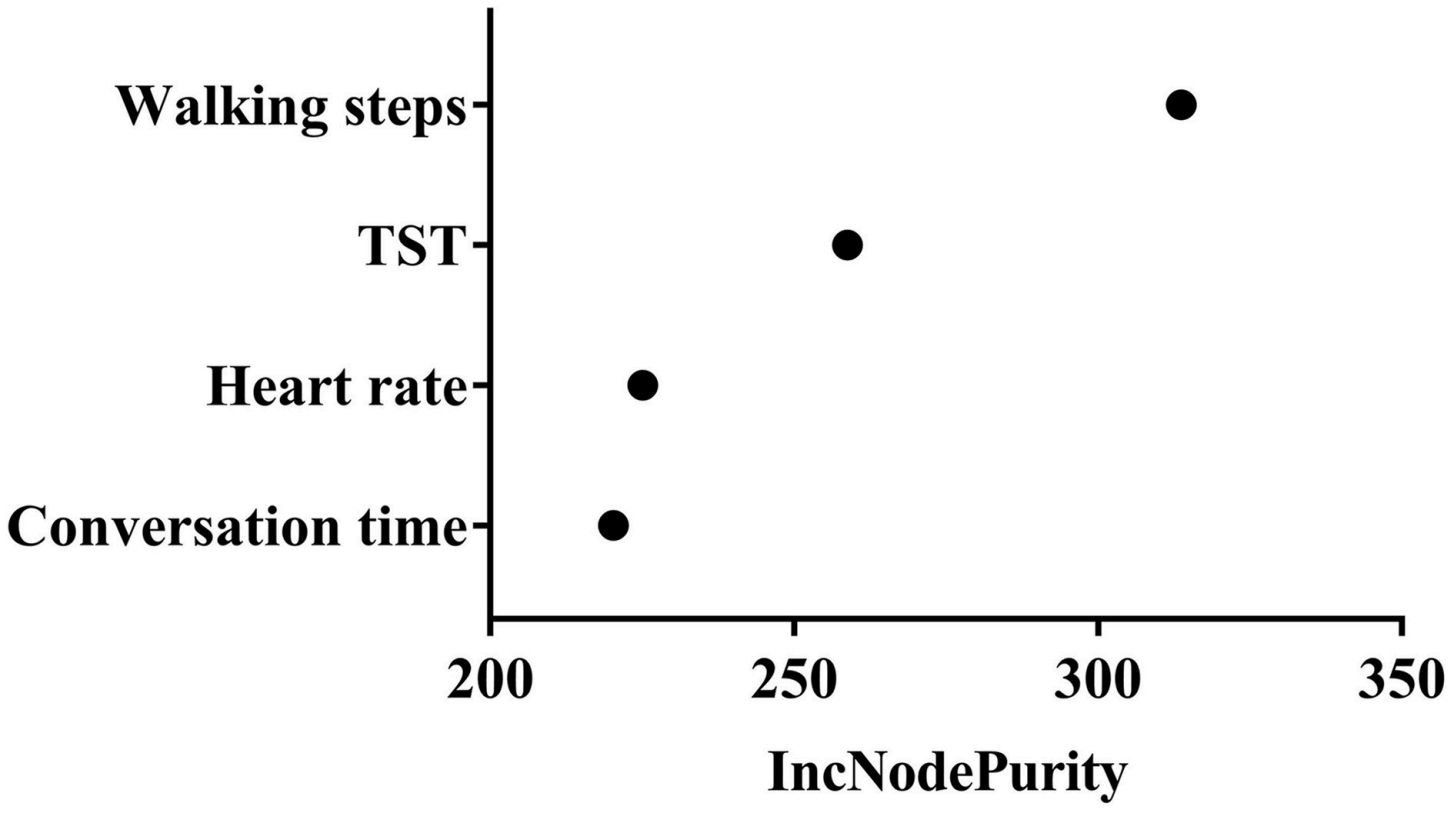
Variable importance measure. The IncNodePurity value of each variable is 313.7 in walking steps, 258.8 in TST, 225.1 in heart rate, and 220.3 in conversation time.

In model 1, three variables, including sleep efficiency, awakening time count, naptime were newly selected and WASO exhibited a < 200% decrease in the estimated parametric values (−440.91%). Therefore, these variables were excluded for next step analysis. Finally, four variables (walking steps, conversation time, TST and heart rate) were included in model 1 for risk and protective factor analysis. The IncNodePurity value of each variable is 313.7 in walking steps, 258.8 in TST, 225.1 in heart rate, and 220.3 in conversation time (Figure 4). The variables regarding physical activity were the most important lifestyle factors associated with cognitive function. In model 2, TST exhibited a sign change in the multiple linear regression models. Therefore, the number of walking steps, conversation time, and heart rate remained significant after the RF regression and confounding factor analysis.

#### Risk and Protective Factor Analysis

**Figure 5:**
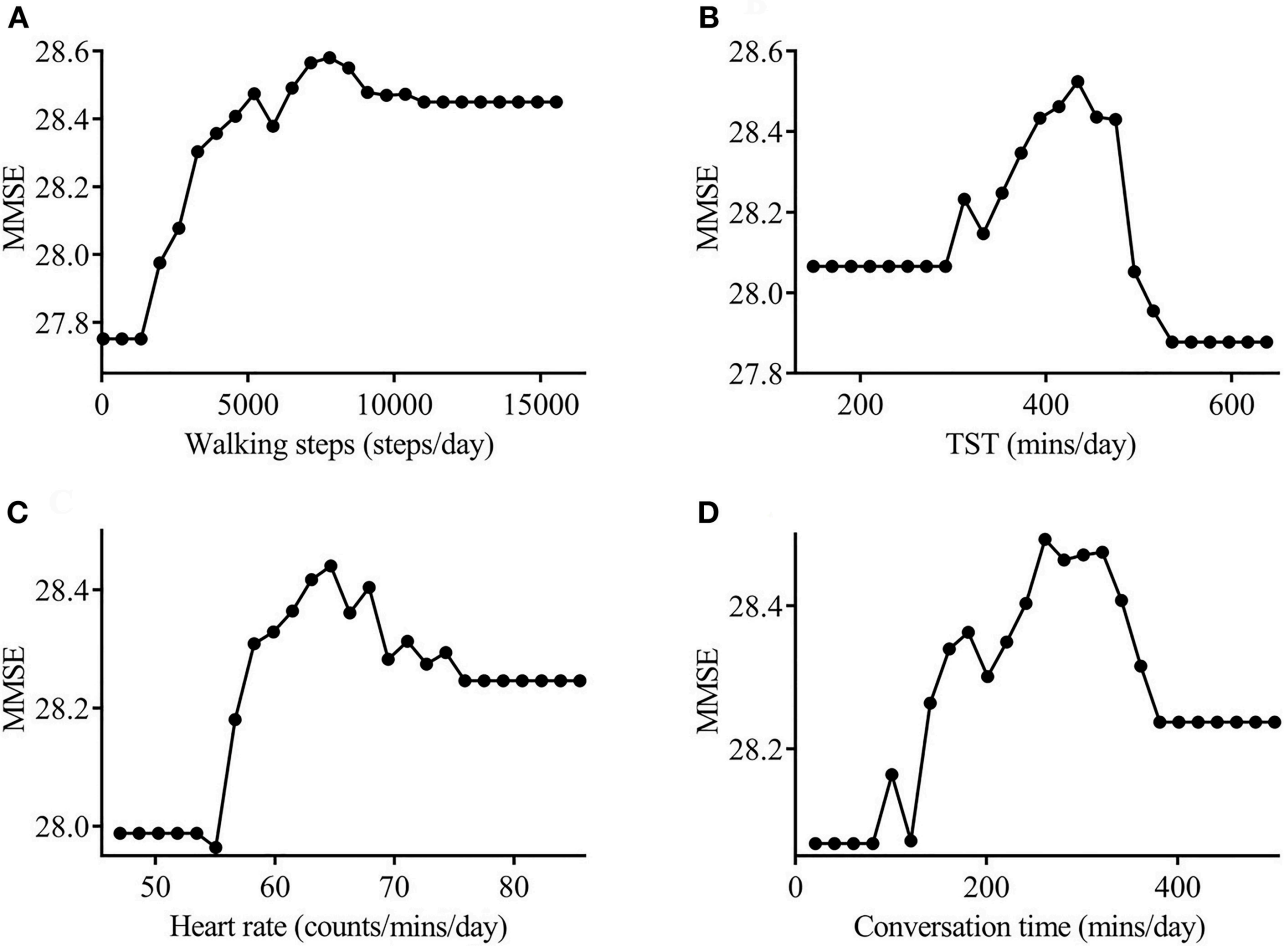
Partial dependency plot for the actinography data. The number of walking steps **(A)**, heart rate **(B)**, and conversation time **(C)** showed a positive correlation with MMSE score and were categorized as protective factors for cognitive function (correlation values: 0.71, 0.547, and 0.396, respectively). TST **(D)** showed a negative correlation with MMSE scores, and was categorized as a risk factor for cognitive function (correlation value; −0.245). The inclination of the graph began to reverse by the boundary of specified threshold in the PDP of conversation time and TST (321.1 min and 495.1 min, respectively). MMSE, Mini-Mental State Examination; TST, tonal sleep time.

**Figure 6:**
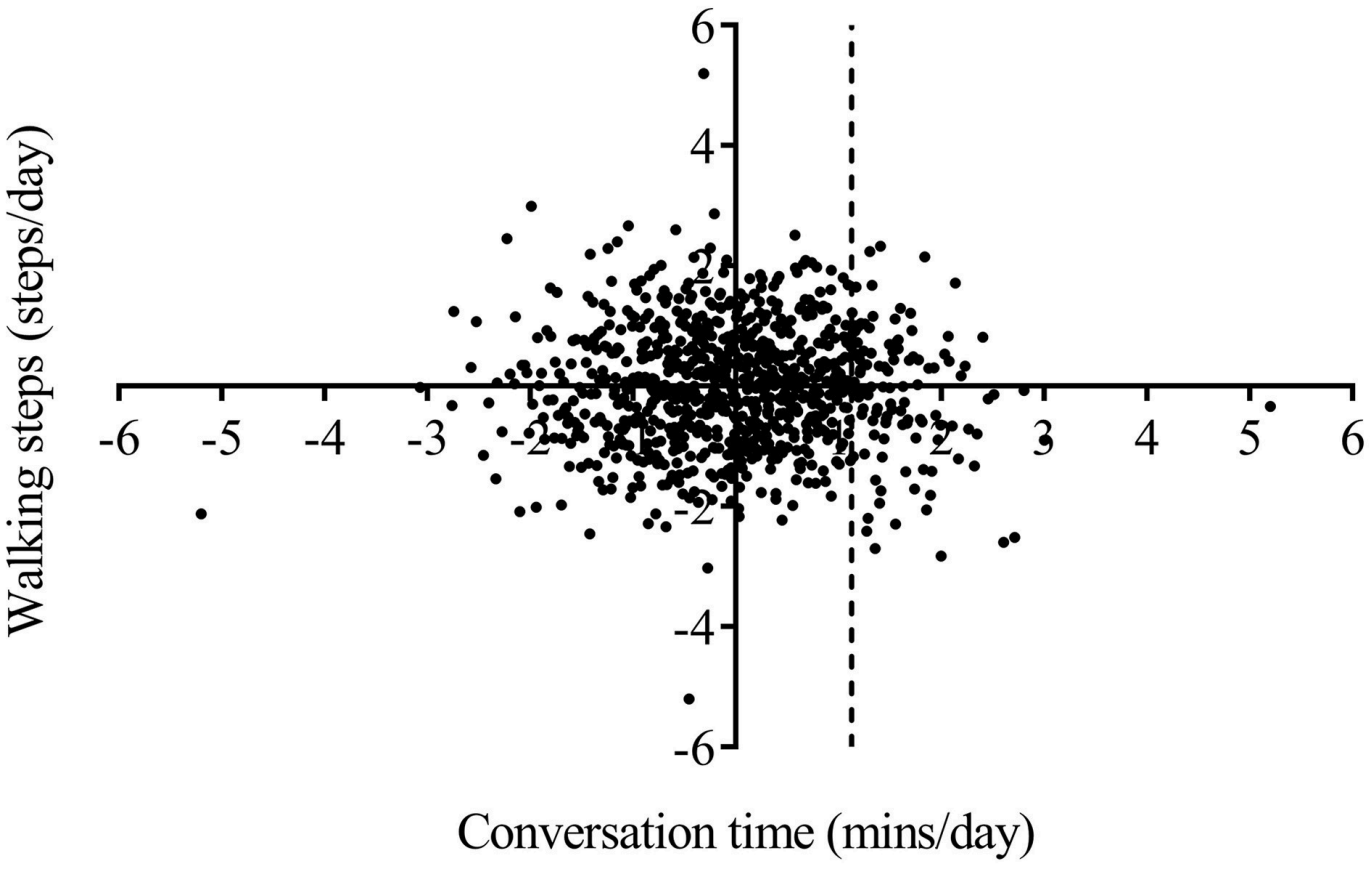
Correlation analysis between the number of walking steps and conversation time. The daily number of walking steps was not correlated with conversation time in participants exhibiting <1.125 min (transformed value, mapping value: 320 min) of conversation, and decreased with increasing conversation time in participants exhibiting more than 1.126 min (transformed value, mapping value: 321 min) of conversation.

Four variables in model 1 were assessed in the protective and risk factor analysis. The number of walking steps, conversation time, and heart rate exhibited positive correlations with MMSE score and were categorized as protective factors for cognitive function, whereas TST was categorized as a risk factor. PDPs of walking steps and heart rate revealed continuously increased MMSE scores. The inclination of the graph, however, began to reverse by the boundary of the specified threshold in the PDP of conversation time and TST (Figure 5). The specified threshold was 321.1 min for conversation time and 495.1 min for TST. Therefore, conversation time and TST were not conclusive risk factors, and appeared to become protective or risk factors according to the length of time. An appropriate duration of sleep for preventing cognitive impairment was 291.6–495.1 min, whereas sleep duration of more than 434.1 min exerted a negative effect on cognitive function. Similarly, an appropriate duration of conversation time for preventing cognitive impairment was 80.8–321.1 min, whereas conversation time of more than 321.1 min exerted a negative effect on cognitive function. In addition, the relationship between conversation time and physical activity was investigated to determine why conversation time beyond the specified threshold was identified as a risk factor. Linear regression analysis was performed after transforming the data to the normal distribution. The results revealed that the walking steps was not correlated with conversation time in participants exhibiting <1.125 min (transformed value, mapping value: 320 min) of conversation (*p* = 0.181, Figure 6), but was negatively correlated with conversation time in participants exhibiting more than 1.126 min (transformed value, mapping value: 321 min) of conversation (*p* = 0.0117, Figure 6).

#### Contribution and Significance of Risk and Protective Variables

To quantify the individual risk and protective variable contributions, multiple linear regression was performed using only the selected four variables (walking steps, TST, heart rate, and conversation time) after transforming the data to the normal distribution (Table 4). The results revealed that walking steps, heart rate, and conversation time were categorized as protective factors for cognitive function (contribution value: 0.4116, 0.1071, and 0.0612, respectively), whereas TST was categorized as a risk factor (contribution value: −0.1128). The walking steps was highly significant.

**Table 4:** Risk and protective variables contribution.

	**Estimate**	**SE**	***t*-value**	***P*-value**
Walking steps (steps/day)	0.4116	0.0722	5.702	1.63e−08*
TST (mins/day)	−0.1128	0.0731	−1.542	0.123
Heart rate (counts/mins/day)	0.1071	0.0726	1.476	0.140
Conversation time (mins/day)	0.0612	0.0721	0.849	0.396

SE, Standard error; TST, Total sleep time; mins, minutes *P < 0.05.

#### Model Prediction Accuracy

The prediction accuracy in the RF model was better than that in the linear regression model (Table 5).

**Table 5:** Model accuracy.

	**Training model**			**Prediction model**		
	***R*^**2**^ value**	**MSE**	**RMSE**	***R*^**2**^ value**	**MSE**	**RMSE**
Linear regression	0.119	3.155	1.776	0.175	2.955	1.719
RF regression	0.775	0.796	0.892	0.759	0.863	0.929

*RF, Random forest; MSE, Mean Squared Error; RMSE, Root mean squared error*.

## Discussion

The present study evaluated lifestyle components, including physical activity, sleep, and conversation, as well as heart rate, using a wearable sensor in a large sample of community-dwelling older people, and constructed a machine learning model to predict cognitive impairment. RF regression analysis adjusted for age, gender, and education years identified four significant variables: walking steps, conversation time, TST, and heart rate. Specifically, walking steps, conversation time, and heart rate remained significant after the RF regression analysis was adjusted for demographic and vascular risk factors. Moreover, the walking steps, conversation time, and heart rate were identified as protective factors for cognitive function, whereas TST was categorized as a risk factor for cognitive function. PDPs of walking steps and heart rate revealed continuously increased MMSE scores. TST and conversation time, however, indicated that the tendency in the PDP graph was reversed at the boundary of a specified threshold. Thus, these variables tended to have a protective effect on cognitive function within a particular range of time, whereas longer periods of time exceeding a particular threshold were risk factors for cognitive function. Because RF regression analysis and PDP graph cannot quantify individual variable contribution, multiple linear regression analysis was performed to quantify individual contribution of the selected variables and also to find its statistical significance. Although the multiple linear regression showed that only the number of walking steps was significant, both RF and linear regression analysis revealed similar results regarding the risk and protective factors for cognition. Moreover, the PDP graph exhibited a reversal at the boundary of a specified threshold in TST and conversation time, which was not detected by linear regression. Therefore, we selected four lifestyle variables related to MMSE scores by RF regression analysis and risk and protective factor analysis of each variable were performed using PDPs. The current findings highlight the importance of physical activity, sleep, and conversation in preventing cognitive impairment among community-dwelling older people.

Numerous studies have examined the beneficial effects of physical activity on cognitive function among older people. A meta-analysis of 15 prospective cohort studies reported protective effects of vigorous exercise against cognitive decline (hazard ratio 0.62, 95% CI 0.54–0.70) (33). Another meta-analysis of 16 studies reported a lower risk ratio of dementia (0.72, 95% CI 0.60–0.86) in the highest physical activity group compared with the lowest physical activity group (34). Moreover, several cross-sectional and prospective studies using actigraphy reported that greater daytime movement was protective against cognitive impairment and dementia (12–15). The results of our cross-sectional study were consistent with previous studies regarding the relationship between total daily movement and cognitive function, and suggested that non-exercise physical activity, such as movement around the house and fidgeting is important for delaying cognitive impairment among older people. Several potential mechanisms have been suggested to explain the beneficial effects of physical activity on cognitive function. Physical activity may reduce brain amyloid deposition and increase brain function by decreasing vascular risk factors, including obesity, hypertension, and diabetes (35–37).

Sleep is important for brain plasticity and memory consolidation (38) and sleep disturbance is a common problem for older people as well as patients with mild cognitive impairment and dementia (8–10, 39). Several cross-sectional or prospective studies reported that shorter and longer sleep duration may be important risk factors for subsequent cognitive impairment (8, 10, 19). The current results indicated that an appropriate duration of sleep was important for delaying cognitive impairment, whereas longer sleep duration (more than 434.1 min) exerted a negative effect on cognitive function in older people. One prospective study of the relationship between sleep duration and the risk of dementia reported that the risk of dementia was increased among individuals with particularly long sleep durations (8 and more than 9 h), compared with those with normal sleep durations (6 and 7 h). These results suggested that longer sleep duration might be a risk factor for cognitive impairment among older people. The sleep-wake cycle is associated with the clearance of brain amyloid-β protein (40), while shorter sleep duration was associated with greater brain amyloid burden on amyloid positron emission tomography (41). However, the mechanisms underlying the relationship between longer sleep duration and dementia remain unclear. Longer sleep duration may increase the risk of dementia, function as an early symptom of dementia, or be associated with sleep disorder-related breathing and smoking habits (10).

The present findings identified daily heart rate as a protective factor for cognitive function among community-dwelling older people. To our knowledge, no previous reports have examined the relationship between heart rate and cognitive function. A previous study reported relationships between resting heart rate, depression, and cognitive impairment in patients with ischemic stroke, and relationships between reduced heart rate variability and cognitive impairment among older women (42–44). Further studies are needed to confirm the influence of heart rate on cognitive function among older people.

Importantly, the current results revealed that conversation time was an important predictive factor for MMSE score. Social isolation and subjective loneliness are increasingly recognized as risk factors for cognitive impairment and dementia among older people (7, 16). A meta-analysis of social activity reported that the risk of developing dementia was increased in individuals with less social participation (relative risk 1.41, 95% CI 1.13–1.75) and less social contact (relative risk 1.57, 95% CI 1.32–1.85) (16). An intervention study reported that active social engagement, including contact with family and friends and positive social support and engagement in leisure activities have beneficial effects for preventing cognitive impairment and dementia (45). In present study, we quantified communication by detecting participants' utterances, using conversation time as a surrogate parameter of social isolation. Few previous studies have examined the relationship between conversation time and cognitive function. Although an appropriate duration of conversation time tended to have a protective effect on cognitive function, we found that longer durations of conversation time (more than 321.1 min) exerted a negative effect on cognitive function among older people. One possible explanation is related to the different effects of conversation time on cognitive function according to the length of time, because longer conversation time was associated with a decreased number of walking steps. Therefore, our results suggest the importance of balance between the duration of conversation and the duration of physical activity. The current results were consistent with previous studies regarding the relationship between communication and cognitive function, highlighting the importance of spending an appropriate proportion of time engaging in conversation. The mechanisms underlying social activity and cognition support the cognitive-reserve hypothesis, which suggests that participation in intellectual, social and physical activities stimulates brain function, resulting in the prevention of dementia (46). In animal studies, mice raised in an enriched environment have been reported to exhibit greater neurogenesis and increased synaptic density, and amyloid precursor protein transgenic mice have been found to exhibit decreased brain amyloid-β deposition in enriched environments (47, 48). Another potential mechanism is that social contact or social support may lead to decreased stress and increased motivation to perform health-related behaviors, resulting in the prevention of dementia (7).

The present study has several limitations that should be considered. First, the study could not determine the causal direction of the association between lifestyle factors and cognitive function because of its cross-sectional design. Second, we were unable to exclude factors influencing cognitive reserve, such as past, or current occupation and engagement in cognitive and social activity, which may affect lifestyle and cognitive function. Third, cognitive function was evaluated only by MMSE and information regarding depression was not collected. The MMSE is a very crude measure of cognition and questionable accuracy for detecting dementia. Although we collected the clinical information to define the present or absence of dementia, the patients with possible dementia could not be excluded completely from participating in the current study. Therefore, further studies assessing a broader range of cognitive domains should be needed to confirm our results. Forth, it is possible that the sound of television viewing or radio listening was detected as conversation due to the detection method based on sound pressure and frequency. Therefore, further studies are needed to improve the reliability of our sensing data. Conversation time may have included sleep or nap time during television viewing or radio listening. The relationship between conversation time and sleep or nap time, however, suggested that the possibility of sleeping being included in the daily conversation time was only 6.4%, which would not be expected to influence the results.

In conclusion, the current study revealed that lifestyle factors such as physical activity, sleep, and social activity were associated with global cognitive function among older people. Physical activity and heart rate were positively associated with cognitive function. Moreover, an appropriate balance between the durations of sleep and conversation appears to be important for cognitive function. These results may contribute to the development of new evidence-based interventions for preventing cognitive impairment and improving health and wellbeing among older people.

## Ethics Statement

This prospective study was conducted in accordance with the Declaration of Helsinki, and was approved by the local ethics committee at Oita University Hospital (UMIN000017442).

## Author Contributions

NK and EM conceived and designed the trial. SN and MK developed the wearable sensor. YA, KY, MI, DH, YS, AN, SU, HF, SI, and TK performed the PET study and conducted the data analysis. AE performed the neuropsychological assessment. KS and MJ helped with the recruitment of participants.

### Conflict of Interest Statement

SN and MK were employed by TDK Corporation. The remaining authors declare that the research was conducted in the absence of any commercial or financial relationships that could be construed as a potential conflict of interest.

## Supplementary Material

Supplemental Figure 1 Linear correlation analysis of walking step. Significant correlation was found between walking steps from wristband sensor and those from video observation (*r* = 0.9869, *p* < 0.0001, Pearson correlation).

Supplemental Figure 2 Linear correlation analysis of sleep duration. Significant correlation was found between sleep duration from wristband sensor and that from video observation (*r* = 0.9995, *p* < 0.0001, Pearson correlation).

Supplemental Figure 3 Linear correlation analysis of conversation time. Significant correlation was found between conversation time from wristband sensor and that from video observation (*r* = 0.8512, *p* < 0.0001, Pearson correlation).

Supplemental Table 1 Results of conversation detection.

Supplemental Table 2 Analysis of false detection rate.

## Abbreviations

BMI: body mass index
CI: confidence interval
MMSE: mini mental state examination
PDP: partial dependency plot
RF: random forest
RMSE: root mean squared error
TST: total sleep time
WASO: wake after sleep onset.

## References

[1] Livingston G Sommerlad A Orgeta V Costafreda SG Huntley J Ames D. Dementia prevention, intervention, and care. Lancet. (2017) 390:2673–734. 10.1016/S0140-6736(17)31363-6 28735855

[2] Daviglus ML Plassman BL Pirzada A Bell CC Bowen PE Burke JR. Risk factors and preventive interventions for Alzheimer disease: state of the science. Arch Neurol. (2011) 68:1185–90. 10.1001/archneurol.2011.100 21555601

[3] Norton S Matthews FE Barnes DE Yaffe K Brayne C. Potential for primary prevention of Alzheimer's disease: an analysis of population-based data. Lancet Neurol. (2014) 13:788–94. 10.1016/S1474-4422(14)70136-X 25030513

[4] Barnes DE Yaffe K. The projected effect of risk factor reduction on Alzheimer's disease prevalence. Lancet Neurol. (2011) 10:819–28. 10.1016/S1474-4422(11)70072-2 21775213 PMC3647614

[5] Clare L Wu YT Teale JC MacLeod C Matthews F Brayne C. Potentially modifiable lifestyle factors, cognitive reserve, and cognitive function in later life: a cross-sectional study. PLoS Med. (2017) 14:e1002259. 10.1371/journal.pmed.1002259 28323829 PMC5360216

[6] Xu W Tan L Wang HF Jiang T Tan MS Tan L. Meta-analysis of modifiable risk factors for Alzheimer's disease. J Neurol Neurosurg Psychiatry. (2015) 86:1299–306. 10.1136/jnnp-2015-310548 26294005

[7] Fratiglioni L Paillard-Borg S Winblad B. An active and socially integrated lifestyle in late life might protect against dementia. Lancet Neurol. (2004) 3:343–53. 10.1016/S1474-4422(04)00767-7 15157849

[8] Benito-León J Bermejo-Pareja F Vega S Louis ED. Total daily sleep duration and the risk of dementia: a prospective population-based study. Eur J Neurol. (2009) 16:990–7. 10.1111/j.1468-1331.2009.02618.x 19473367

[9] Oosterman JM van Someren EJ Vogels RL Van Harten B Scherder EJ. Fragmentation of the rest-activity rhythm correlates with age-related cognitive deficits. J Sleep Res. (2009) 18:129–35. 10.1111/j.1365-2869.2008.00704.x 19250179

[10] Benito-León J Louis ED Villarejo-Galende A Romero JP Bermejo-Pareja F. Long sleep duration in elders without dementia increases risk of dementia mortality (NEDICES). Neurology. (2014) 83:1530–7. 10.1212/WNL.0000000000000915 25253755 PMC4222849

[11] Blackwell T Yaffe K Ancoli-Israel S Redline S Ensrud KE Stefanick ML. Association of sleep characteristics and cognition in older community-dwelling men: the MrOS sleep study. Sleep. (2011) 34:1347–56. 10.5665/SLEEP.1276 21966066 PMC3174836

[12] Schlosser Covell GE Hoffman-Snyder CR Wellik KE Woodruff BK Geda YE Caselli RJ Physical activity level and future risk of mild cognitive impairment or dementia: a critically appraised topic. Neurologist. (2015) 19:89–91. 10.1097/NRL.0000000000000013 25692517

[13] Buchman AS Boyle PA Yu L Shah RC Wilson RS Bennett DA. Total daily physical activity and the risk of AD and cognitive decline in older adults. Neurology. (2012) 78:1323–9. 10.1212/WNL.0b013e3182535d35 22517108 PMC3335448

[14] Barnes DE Blackwell T Stone KL Goldman SE Hillier T Yaffe K Study of osteoporotic fractures. cognition in older women: the importance of daytime movement. J Am Geriatr Soc. (2008) 56:1658–64. 10.1111/j.1532-5415.2008.01841.x 18662201 PMC2680379

[15] Falck RS Landry GJ Best JR Davis JC Chiu BK Liu-Ambrose T. Cross-sectional relationships of physical activity and sedentary behavior with cognitive function in older adults with probable mild cognitive impairment. Phys Ther. (2017) 97:975–84. 10.1093/ptj/pzx074 29029554 PMC5803762

[16] Kuiper JS Zuidersma M Oude Voshaar RC Zuidema SU van den Heuvel ER Stolk RP. Social relationships and risk of dementia: a systematic review and meta-analysis of longitudinal cohort studies. Ageing Res Rev. (2015) 22:39–57. 10.1016/j.arr.2015.04.006 25956016

[17] Biddle DJ Naismith SL Griffiths KM Christensen H Hickie IB Glozier NS. Associations of objective and subjective sleep disturbance with cognitive function in older men with comorbid depression and insomnia. Sleep Health. (2017) 3:178–83. 10.1016/j.sleh.2017.03.007 28526255

[18] Cross N Terpening Z Rogers NL Duffy SL Hickie IB Lewis SJ Napping in older people “at risk” of dementia: relationships with depression, cognition, medical burden, and sleep quality. J Sleep Res. (2015) 24:494–502. 10.1111/jsr.12313 26096839

[19] Spira AP Stone KL Redline S Ensrud KE Ancoli-Israel S Cauley JA. Actigraphic sleep duration and fragmentation in older women: associations with performance across cognitive domains. Sleep. (2017) 40. 10.1093/sleep/zsx073 28472447 PMC5806540

[20] Diem SJ Blackwell TL Stone KL Yaffe K Tranah G Cauley JA Measures of sleep-wake patterns and risk of mild cognitive impairment or dementia in older women. Am J Geriatr Psychiatry. (2016) 24:248–58. 10.1016/j.jagp.2015.12.002 26964485 PMC4807599

[21] Blackwell T Yaffe K Ancoli-Israel S Schneider JL Cauley JA Hillier TA. Poor sleep is associated with impaired cognitive function in older women: the study of osteoporotic fractures. J Gerontol A Biol Sci Med Sci. (2006) 61:405–10. 10.1093/gerona/61.4.405 16611709

[22] Breiman L Cutler A Random Forests. (2005). Available online at: https://www.stat.berkeley.edu/users/breiman/RandomForests/

[23] Obermeyer Z Emanuel EJ. Predicting the future: big data, machine learning, and clinical medicine. New Eng J Med. (2016) 375:1216–9. 10.1056/NEJMp1606181 27682033 PMC5070532

[24] Bhagyashree SIR Nagaraj K Prince M Fall CHD Krishna M. Diagnosis of dementia by machine learning methods in epidemiological studies: a pilot exploratory study from South India. Soc Psychiatry Psychiatr Epidemiol. (20189 53:77–86. 10.1007/s00127-017-1410-0 28698926 PMC6138240

[25] Lee SK Son YJ Kim J Kim HG Lee JI Kang BY. Prediction model for health-related quality of life of elderly with chronic diseases using machine learning techniques. Healthc Inform Res. (2014) 20:125–34. 10.4258/hir.2014.20.2.125 24872911 PMC4030056

[26] Casanova R Saldana S Lutz MW Plassman BL Kuchibhatla M Hayden KM. Investigating predictors of cognitive decline using machine learning. J Gerontol B Psychol Sci Soc Sci. (2018) 10.1093/geronb/gby054 [Epub ahead of print]. 29718387 PMC7205421

[27] Ngandu T Lehtisalo J Solomon A Levälahti E Ahtiluoto S Antikainen R. A 2 year multidomain intervention of diet, exercise, cognitive training, and vascular risk monitoring versus control to prevent cognitive decline in at-risk elderly people (FINGER): a randomised controlled trial. Lancet. (2015) 385:2255–63. 10.1016/S0140-6736(15)60461-5 25771249

[28] American Psychiatric Association Diagnostic and Statistical Manual of Mental Disorders, 5th ed. DSM−5. Arlington, VA: American Psychiatric Association (2013).

[29] Breiman L Bagging predictors. Machine Learning. (1996) 24:123–40. 10.1007/BF00058655

[30] R Core Team R: a Language and Environment for Statistical Computing. Vienna, Austria: R Foundation for Statistical Computing (2018). Available online at: https://www.R-project.org/

[31] Pourhoseingholi MA Baghestani AR Vahedi M. How to control confounding effects by statistical analysis. Gastroenterol Hepatol Bed Bench. (2012) 5:79–83. 24834204 PMC4017459

[32] Hastie T Tibshirani Robert T Friedman J The Elements of Statistical Learning. Data Mining, Inference, and Prediction, 2nd ed. Berlin: Springer (2009).

[33] Sofi F Valecchi D Bacci D Abbate R Gensini GF Casini A. Physical activity and risk of cognitive decline: a meta-analysis of prospective studies. J Intern Med. (2011)269:107–17. 10.1111/j.1365-2796.2010.02281.x 20831630

[34] Hamer M Chida Y. Physical activity and risk of neurodegenerative disease: a systematic review of prospective evidence. Psychol Med. (2009) 39:3–11. 10.1017/S0033291708003681 18570697

[35] Adlard PA Perreau VM Pop V Cotman CW. Voluntary exercise decreases amyloid load in a transgenic model of Alzheimer's disease. J Neurosci. (2005) 25:4217–21. 10.1523/JNEUROSCI.0496-05.2005 15858047 PMC6725122

[36] Laurin D Verreault R Lindsay J MacPherson K Rockwood K. Physical activity and risk of cognitive impairment and dementia in elderly persons. Arch Neurol. (2001) 58:498–504. 10.1001/archneur.58.3.498 11255456

[37] Chieffi S Messina G Villano I Messina A Valenzano A Moscatelli F. Neuroprotective effects of physical activity: evidence from human and animal studies. Front Neurol. (2017) 8:188. 10.3389/fneur.2017.00188 28588546 PMC5439530

[38] Abel T Havekes R Saletin JM Walker MP. Sleep, plasticity, and memory from molecules to whole-brain networks. Curr Biol. (2013) 23:R774–88. 10.1016/j.cub.2013.07.025 24028961 PMC4263505

[39] da Silva RA Sleep disturbances and mild cognitive impairment: a review. Sleep Sci. (2015) 8:36–41. 10.1016/j.slsci.2015.02.001 26483941 PMC4608881

[40] Kang JE Lim MM Bateman RJ Lee JJ Smyth LP Cirrito JR. Amyloid-beta dynamics are regulated by orexin and the sleep-wake cycle. Science. (2009) 326:1005–7. 10.1126/science.1180962 19779148 PMC2789838

[41] Spira AP Gamaldo AA An Y Wu MN Simonsick EM Bilgel M. Self-reported sleep and β-amyloid deposition in community-dwelling older adults. JAMA Neurol. (2013) 70:1537–43. 10.1001/jamaneurol.2013.4258 24145859 PMC3918480

[42] Böhm M Cotton D Foster L Custodis F Laufs U Sacco R. Impact of resting heart rate on mortality, disability and cognitive decline in patients after ischaemic stroke. Eur Heart J. (2012) 33:2804–12. 10.1093/eurheartj/ehs250 22922507

[43] Tessier A Sibon I Poli M Audiffren M Allard M Pfeuty M. Resting heart rate predicts depression and cognition early after ischemic stroke: a pilot study. J Stroke Cerebrovasc Dis. (2017) 26:2435–41. 10.1016/j.jstrokecerebrovasdis.2017.05.040 28652061

[44] Kim DH Lipsitz LA Ferrucci L Varadhan R Guralnik JM Carlson MC. Association between reduced heart rate variability and cognitive impairment in older disabled women in the community: Women's Health and Aging Study I. J Am Geriatr Soc. (2006) 54:1751–7. 10.1111/j.1532-5415.2006.00940.x 17087704 PMC2276586

[45] Marioni RE Proust-Lima C Amieva H Brayne C Matthews FE Dartigues JF. Social activity, cognitive decline, and dementia risk: a 20-year prospective cohort study. BMC Public Health. (2015) 15:1089. 10.1186/s12889-015-2426-6 26499254 PMC4619410

[46] Stern Y. Cognitive reserve in ageing and Alzheimer's disease. Lancet Neurol. (2012) 11:1006–12. 10.1016/S1474-4422(12)70191-6 23079557 PMC3507991

[47] van Praag H Kempermann G Gage FH Running increases cell proliferation and neurogenesis in the adult mouse dentate gyrus. Nat Neurosci. (1999) 2:266–70. 10.1038/6368 10195220

[48] Lazarov O Robinson J Tang YP Hairston IS Korade-Mirnics Z Lee VM. Environmental enrichment reduces Abeta levels and amyloid deposition in transgenic mice. Cell. (2005) 120:701–13. 10.1016/j.cell.2005.01.015 15766532

