# Supplementary figures and images for: Modifiable Lifestyle Factors and Cognitive Function in Older People: A Cross-Sectional Observational Study
Source: Front Neurol. 2019 Apr 24;10:401. doi: 10.3389/fneur.2019.00401 (PMC6491512; doi:10.3389/fneur.2019.00401)

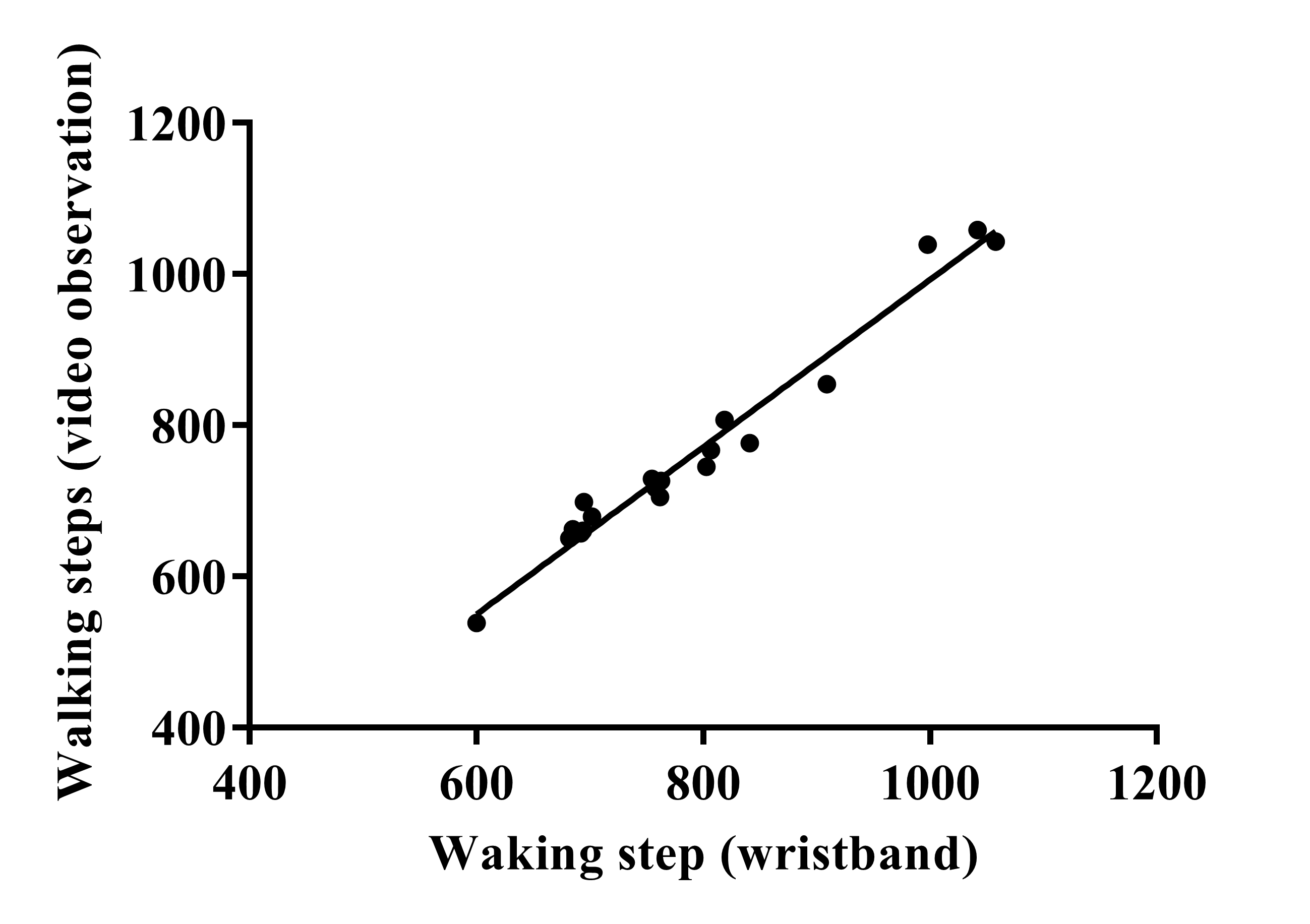

Supplement: Supplemental Figure 1 — Linear correlation analysis of walking step. Significant correlation was found between walking steps from wristband sensor and those from video observation (r = 0.9869, p < 0.0001, Pearson correlation). [file Image_1.TIF]

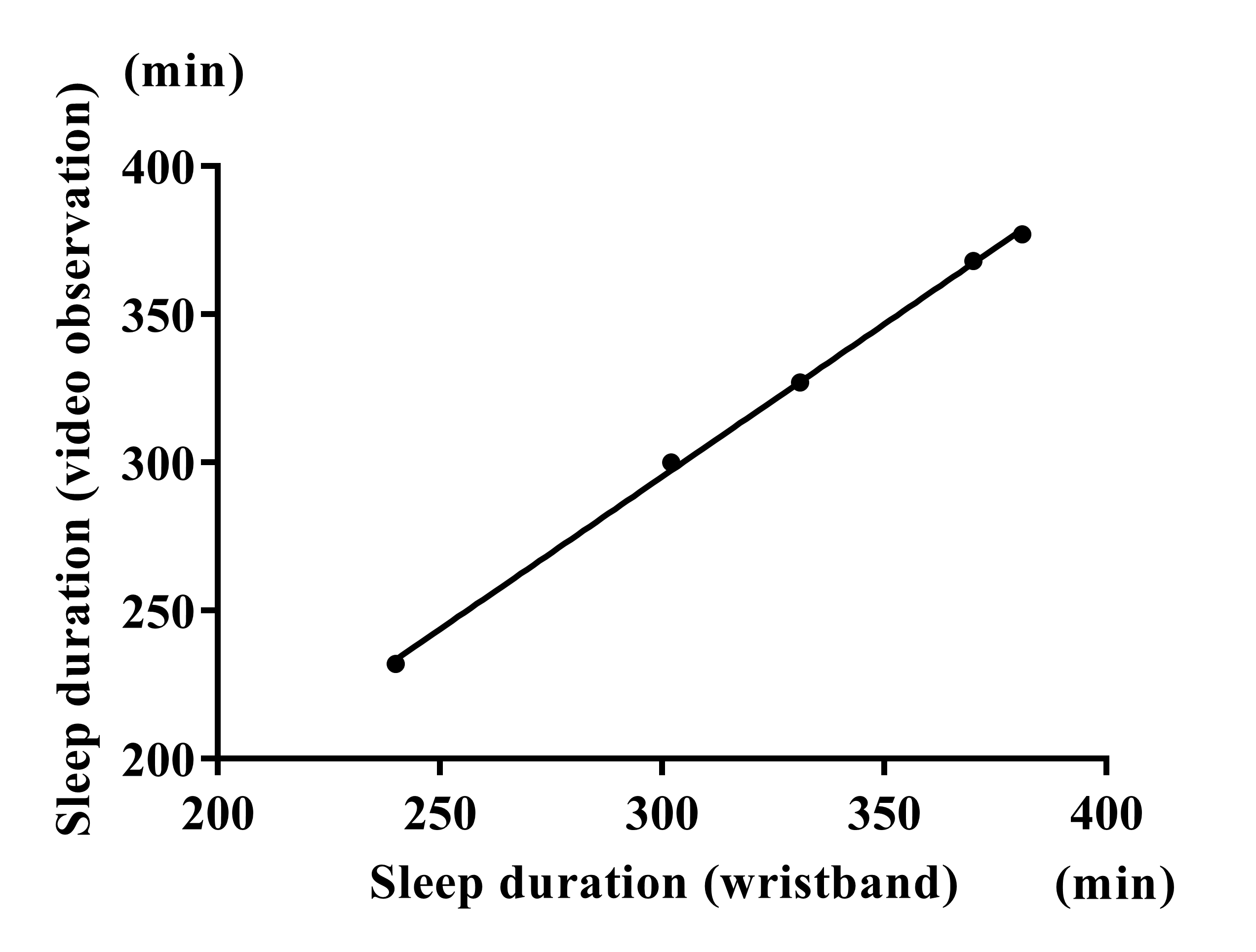

Supplement: Supplemental Figure 2 — Linear correlation analysis of sleep duration. Significant correlation was found between sleep duration from wristband sensor and that from video observation (r = 0.9995, p < 0.0001, Pearson correlation). [file Image_2.TIF]

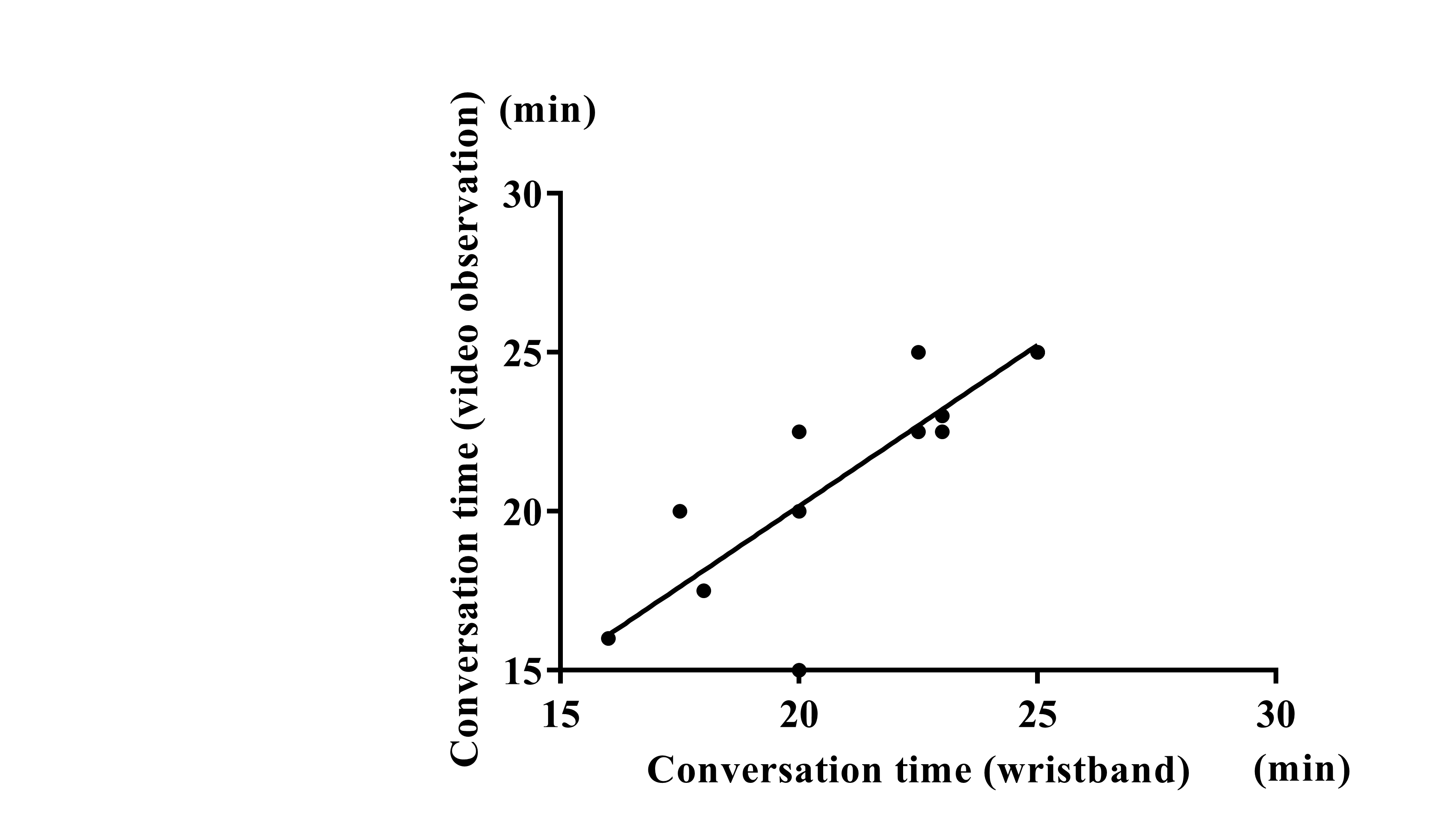

Supplement: Supplemental Figure 3 — Linear correlation analysis of conversation time. Significant correlation was found between conversation time from wristband sensor and that from video observation (r = 0.8512, p < 0.0001, Pearson correlation). [file Image_3.TIF]
