# Supplementary material for: Modifiable Lifestyle Factors and Cognitive Function in Older People: A Cross-Sectional Observational Study
Source: Front Neurol. 2019 Apr 24;10:401. doi: 10.3389/fneur.2019.00401 (PMC6491512; doi:10.3389/fneur.2019.00401)
Supplement: Supplemental Table 1 — Results of conversation detection. [file Table_1.DOCX]

**Supplemental table 1. Results of conversation detection**

|  | Self-reported conversation time (min) | |
| --- | --- | --- |
|  | True conversation  (T) | No or false conversation (F) |
| Sound defined by device as conversation (P) | 367 (TP) | 159 (FP) |
| Sound defined by device as non-conversation (N) | 107 (TN) | 2427 (FN) |

Precision (P) = TP / (TP + FP), Recall (R) = TP / (TP + FN), F-Measure (F) = 2PR / (P + R)
