# Supplementary material for: Modifiable Lifestyle Factors and Cognitive Function in Older People: A Cross-Sectional Observational Study
Source: Front Neurol. 2019 Apr 24;10:401. doi: 10.3389/fneur.2019.00401 (PMC6491512; doi:10.3389/fneur.2019.00401)
Supplement: Supplemental Table 2 — Analysis of false detection rate. [file Table_2.DOCX]

**Supplemental table 2. Analysis of false detection rate**

|  | FP | TN | FDR |
| --- | --- | --- | --- |
| Television | 36 | 89 | 0.288 |
| Noise during commuting | 15 | 250 | 0.057 |
| Noise during office work | 89 | 739 | 0.107 |

T: True conversation. F: No or false conversation.

P: Device defined sound as conversation. N: Device defined sound as non-conversation.

False Detection Ratio (FDR) = FP / (FP + TN).
